# Advancing Women Leaders in Global Health: Getting to Solutions

**DOI:** 10.29024/aogh.2384

**Published:** 2018-11-05

**Authors:** Cheryl A. Moyer, Nauzley C. Abedini, Jessica Youngblood, Zohray Talib, Tanvi Jayaraman, Mehr Manzoor, Heidi J. Larson, Patricia J. Garcia, Agnes Binagwaho, Katherine S. Burke, Michele Barry

**Affiliations:** 1 Global REACH and the Department of Learning Health Sciences, University of Michigan, 1111 Catherine Street, Ann Arbor, MI, 48109, US; 2 National Clinician Scholars Program and Division of Hospital Medicine, University of Michigan. UH South Unit 4, 1500 East Medical Center Drive, Ann Arbor, MI 48109, US; 3 George Washington University, 1311 Gatesmeadow Way, Reston VA, 20194, US; 4 Stanford Center for Innovation in Global Health, Stanford University, 291 Campus Drive, Stanford, CA 94305, US; 5 Women in Global Health and Tulane University, 1440 Canal Street, New Orleans, Louisiana, 70112, US; 6 London School of Hygiene and Tropical Medicine, Keppel St, Bloomsbury, London, WE1E, 7HT, UK; 7 Cayetano Heredia University, School of Public Health, Av Honorio Delgado 430 SMP Lima 31, PE; 8 University of Global Health Equity, Kigali Heights, plot 772, KG 7 Ave, 5th Kigali, RW

## Abstract

**Background:**

Women comprise 75% of the health workforce in many countries and the majority of students in academic global health tracks but are underrepresented in global health leadership. This study aimed to elucidate prevailing attitudes, perceptions, and beliefs of women and men regarding opportunities and barriers for women’s career advancement, as well as what can be done to address barriers going forward.

**Methods:**

This was a convergent mixed-methods, cross-sectional, anonymous, online study of participants, applicants, and those who expressed an interest in the Women Leaders in Global Health Conference at Stanford University October 11–12, 2017. Respondents completed a 26-question survey regarding beliefs about barriers and solutions to addressing advancement for women in global health.

**Findings:**

405 participants responded: 96.7% were female, 61.6% were aged 40 or under, 64.0% were originally from high-income countries. Regardless of age or country of origin, leading barriers were: lack of mentorship, challenges of balancing work and home, gender bias, and lack of assertiveness/confidence. Proposed solutions were categorized as individual or meta-level solutions and included senior women seeking junior women for mentorship and sponsorship, junior women pro-actively making their desire for leadership known, and institutions incentivizing mentorship and implementing targeted recruitment to improve diversity of leadership.

**Interpretation:**

This study is the first of its kind to attempt to quantify both the barriers to advancement for women leaders in global health as well as the potential solutions. While there is no shortage of barriers, we believe there is room for optimism. A new leadership paradigm that values diversity of thought and diversity of experience will benefit not only the marginalized groups that need to gain representation at the table, but ultimately the broader population who may benefit from new ways of approaching long-standing, intractable problems.

## Introduction

Discussions around equity for women in the workforce and positions of power are gathering momentum worldwide [1,2,3,4,5]. Women comprise as much as 75% of the health workforce in many countries and make up a large majority of students in academic global health tracks [6], but are underrepresented in leadership. Women hold 8 of 34 World Health Organization executive board positions, and fewer than 1 in 4 global health leadership positions at the top 50 US medical schools [7,8]. Globally, 31% of ministers of health are women (Mehr and Ghant, unpublished, 2017), and in Africa, only 25% of ministers of health are women [6]. Of the 27 companies comprising the health sector of the global *Fortune 500*, only one is led by a woman [9].

The 2017 Women Leaders in Global Health conference at Stanford University gathered more than 400 leaders from 68 countries, representing more than 250 institutions and organizations, to focus on issues of gender parity in global health leadership, building on efforts by Women in Global Health, Research in Gender and Ethics, and others [10].

Given the inattention to gender issues in the literature on human resources for health, it is not surprising that little data exist on women in global health. This study aimed to collect qualitative and quantitative data from both women and men in the global health field on knowledge, attitudes, perceptions, and beliefs regarding opportunities and barriers for women’s career advancement and seeking leadership roles in global health, as well as what can be done to address these barriers going forward. Specifically, our research questions were: 1) What are perceived barriers to women’s advancement into positions of global health leadership? 2) What are perceptions regarding the role of gender bias in impeding career growth? 3) What are suggested solutions and pathways forward to improve female representation in positions of global health leadership?

## Methods

This was a convergent mixed-methods, cross-sectional, anonymous, online study of participants, applicants, and those who expressed an interest in the Women Leaders in Global Health Conference held at Stanford University October 11–12, 2017. Respondents were solicited via email from the 783 individuals who expressed an interest in or applied to attend the conference and by distributing a link to the 424 conference participants. Conference participants were encouraged to distribute the survey link to colleagues, and a link to the survey was posted on the Stanford Global Health website and the Women in Global Health websites from October 12 to December 27, 2017. Due to the anonymous nature of the survey and the ability of individuals to forward the link, we do not know the total denominator for potential respondents, and thus we cannot calculate a precise response rate.

The survey was developed through a review of the KPMG Women’s Leadership Study survey tool (KPMG.com/WomensLeadership), expert consultation with the 59 Women Leaders in Global Health Steering Committee, and an iterative process of item development, testing, and revision. The final tool included 26 questions, including 22 quantitative and 4 open-ended qualitative questions. (See Appendix.) Quantitative questions were designed to document perceptions of the current situation in global health leadership, whereas qualitative questions were designed to delve deeper into those perceptions and identify potential solutions.

Data were collected using Survey Monkey (www.surveymonkey.com), and respondents could complete the survey online at the time and location of their choice. The introduction included consent language informing participants that the survey was voluntary and data were being collected anonymously. All data were automatically collected and uploaded to the SurveyMonkey.com servers. Data were downloaded into Microsoft Excel (Redmond, WA), cleaned, and then imported into Stata 15.1 for statistical analysis or NVivo 10.0 for qualitative analysis.

*Quantitative data* included two main sets of outcome variables. The first was how respondents ranked a list of potential barriers to women’s advancement in global health, and the second was whether respondents believed that gender bias had impacted their own career growth in global health. Respondents were asked to rank potential barriers as “one of the most important barriers”, “somewhat important”, or “not at all important”, and choose three barriers they thought to be most important. Gender bias was assessed using reactions to the statement, “I personally feel like gender bias has affected my career growth in global health”. Response options were “strongly disagree”, “somewhat disagree”, “neither agree nor disagree”, “somewhat agree”, and “strongly agree”. Somewhat and strongly agree were combined to create a dummy variable in which 1 = bias impacted career growth and 0 = bias did not impact career growth.

Dependent variables included age, gender, country of origin (low- or middle-income (LMIC) versus high-income (HIC)), country of current residence (LMIC vs. HIC), race/ethnicity, marital status, employment status, career stage, type of employer, and leadership role (see Table 1).

**Table 1:** Demographics of Survey Participants (N = 405).

	N(%)

Age:	
30 or under	103 (25.5)
31–40	146 (36.1)
41–50	80 (19.8)
51–60	50 (12.4)
61–70	19 (4.7)
71 or older	6 (1.5)
Gender:	
Female	392 (96.7)
Male	13 (3.2)
Country of origin:	
Low- or middle-income country	145 (36.0)
High-income country	258 (64.0)
Country of current residence:	
Low- or middle-income country	96 (23.8)
High-income country	307 (76.2)
Race/Ethnicity:	
African	72 (17.9)
Asian	70 (17.4)
Hispanic	18 (4.5)
White	202 (50.1)
Mixed	27 (6.7)
Other	14 (3.5)
Marital Status:	
Married/domestic partnership	220 (54.6)
Single (never married)	146 (36.2)
Divorced/widowed/separated	37 (9.2)
Employment status:	
Working full-time	337 (83.6)
Working part-time	34 (8.4)
Not currently working	32 (7.9)
Career stage:	
Early career	177 (43.8)
Mid career	170 (42.1)
Late career	57 (14.1)
Type of employer:	
Academia	197 (48.8)
Private/for-profit	34 (8.4)
Private/not-for-profit	116 (28.7)
Public sector/government	35 (8.7)
Other	22 (5.5)
Leadership role:	
In a position of leadership	147 (36.4)
Aspire to a position of leadership	241 (59.6)
Do not aspire to leadership position	16 (4.0)
Attended the Women Leaders in Global Health Conference:	
Attended in person	221 (54.6)
Attended via livestream	42 (10.4)
Did not attend	142 (35.0)

Descriptive statistics were calculated, and bivariate analyses compared HIC respondents with respondents from LMICs. Bivariate analysis was also conducted comparing respondents aged 40 and under with those respondents aged 41 and over, as well as those who attended the conference and those who didn’t. Multivariate logistic regression analysis was conducted to determine the factors associated with perceiving gender bias had impacted one’s global health career growth. Given multiple comparisons, a p value of 0.01 was taken to be statistically significant.

Per the *Qualitative Data*, all qualitative responses were read several times by two of the authors (CAM, NCA) and preliminary themes were identified. A codebook was developed, and the same two authors hand-coded printed transcripts. Hand-coding was double checked and imported into NVivo 10.0 by a third coder (JY). In the case of discrepancies, the three coders discussed the passages in question until consensus could be reached regarding the appropriate code. There were no discrepancies that could not be resolved. Once all data were coded, the codebook and original themes were revisited and discussed at length to identify overarching themes emerging from the data. Themes were then compared against quantitative findings to determine where qualitative data provided additional or contradictory information. The overarching theme of “potential solutions” was further sub-coded into individual solutions and meta-level solutions.

*Ethical review*: This study was reviewed and determined to eligible for non-regulated status by the University of Michigan Institutional Review Board (HUM00135881).

## Results

Table 1 illustrates sample demographics. A total of 405 participants responded, 96.7% were female, 61.6% were aged 40 or under, 64.0% were originally from HICs, and 76.2% currently lived in an HIC. Nearly two-thirds of respondents attended the conference in person (N = 241, 59.6%) or via livestream (N = 42, 10.4%), with 35% of respondents not attending (N = 142, 35.0%). None of the results presented varied by whether respondents had attended the conference or not.

### Perceived barriers to advancement

Table 2 illustrates perceived barriers to advancement, stratified by current residence in an HIC versus LMIC. There was no statistically significant difference regarding agreement with statement that “Women face unique barriers to advancing to positions of global health leadership compared to men.”

**Table 2:** Perceived barriers to advancement in global health, stratified by respondents who currently live in high-income versus low- and middle-income country.

	Overall N (%) who agreed/strongly agreed	High-Income Country Residents (N = 264) N (%) who agreed/strongly agreed	Low- and Middle-Income Country Residents (N = 88) N (%) who agreed/strongly agreed	Chi Square (P value)

Women face unique barriers to advancing to positions of global health leadership compared to men (N = 352)	317 (89.5)	242 (91.7)	73 (83.0)	12.3 (0.015)
I personally feel like gender bias has affected my career growth in global health (N = 352)	209 (59.0)	153 (57.9)	54 (61.3)	3.7 (0.44)
	**Overall N (%) who marked as one of the most important barriers**	**High-Income Country Respondents N (%) who marked as one of the most important barriers**	**Low- and Middle-Income Country Respondents N (%) who marked as one of the most important barriers**	**Chi Square (P value)**

Lack of mentorship (N = 336)	188 (56.0)	130 (52.2)	57 (67.1)	6.5 (0.04)
Balancing work and home (N = 320)	167 (52.2)	117 (49.2)	49 (60.5)	3.4 (0.184)
Gender bias in home country (N = 320)	161 (50.3)	119 (49.6)	42 (52.5)	0.21 (0.896)
Lack of female mentors (N = 322)	135 (41.9)	98 (40.5)	37 (46.8)	1.01 (0.60)
Lack of assertiveness/confidence (N = 315)	117 (37.1)	85 (36.5)	32 (39.5)	1.4 (0.478)
Lack of opportunities (N = 312)	109 (34.9)	69 (29.7)	40 (50.6)	**11.6 (0.003)**
Lack of funding for meetings and networking (N = 313)	104 (33.2)	60 (26.0)	43 (53.8)	**21.9 (<0.001)**
Gender bias in partner country (N = 309)	97 (31.4)	74 (32.0)	23 (29.9)	5.25 (0.072)
Inadequate pay (N = 312)	97 (31.1)	65 (28.0)	31 (39.7)	4.4 (0.111)
Safety concerns (N = 302)	72 (23.7)	37 (16.4)	35 (45.5)	**27.2 (<0.001)**
Travel requirements (N = 302)	68 (22.5)	40 (17.8)	27 (35.5)	**10.4 (0.006)**
Work Load (N = 305)	64 (21.0)	32 (14.0)	31 (40.8)	**30.6 (<0.001)**
Lack of training (N = 294)	53 (18.0)	28(12.7)	25 (34.3)	**21.9 (<0.001)**

Overall, quantitative data suggest that the leading barriers to advancement (in descending order) were lack of mentorship, challenges of balancing work and home, gender bias in respondent’s home country, lack of female mentors, and lack of assertiveness/confidence. These barriers were universally agreed on by respondents from both HICs and LMICs. However, respondents from LMICs rated lack of opportunities, lack of funding for meetings and networking, safety concerns, travel requirements, work load, and lack of training significantly higher than their HIC counterparts.

When these same barriers were stratified by age (40 and younger and 41+ years old), there was no significant difference between age groups across any of the barriers (data not shown).

Qualitative data corroborated the quantitative findings, yet suggested a slightly different ordering of the most prominent barriers. When ranked by the number of times an issue was mentioned in the qualitative data, the most prominent barriers (in descending order) were systemic/cultural factors including gender bias (mentioned 377 times), the need for mentorship/sponsorship (341 mentions), needing more support (185 mentions), issues surrounding work/life balance (137 mentions), and women’s own characteristics (136 mentions).

Two additional issues were raised in the qualitative data that were not found in the quantitative data: first, that women of color and women from other marginalized groups face a double burden; and second, that women and men often have very different leadership styles, and the male model is considered normative.

Table 3 provides illustrative quotes for each thematic area.

**Table 3:** Illustrative quotes.


**Systemic/cultural factors**
“Lingering norms about what women can/can’t do and how they should/shouldn’t act in professional situations hold women back and subtly but powerfully discourage them from pursuing or being considered for leadership roles.”
“Implicit bias still exists significantly …(regarding) how a woman is supposed to run her life and behave compared to men. If we speak up, we are still treated like bitches vs. men are simply (seen as) assertive and speaking their mind. And women are… (judged by) whether or not they have children and the spouse. Either way we can’t win. Much easier for men. I don’t know that this is specific to global health though.”
**Mentorship and sponsorship**
“While there are many women working in lower level positions in global health organizations and government positions related to global health, there are many fewer women working in high level positions, which means there are fewer mentors available to help us navigate the different things that make a global health career more challenging for women. Having mentors that understand what it means to not be respected by male colleagues and men in upper level positions, who encourage you to speak up, who understand that there are different safety concerns to consider when working in the field in any country for women is invaluable.”
**Need for Support**
“There simply is not enough support from men. Men and the socialization of men to not actively integrate, support, or stand up for their women counter-parts has shaped an ethos where women’s voices are consistently unheard.”
“(We need more) programs that support mid-career women. Most new programs are aimed at early-career or students, which is great, but that has to continue through mid-career to get women into the C-suite.”
**Role Conflict and work/life balance**
“Women will do anything for their families. If faced with a trying situation… career suffers.”
“Our biology for making and rearing babies who become global citizens, while truly unique and beautiful, is so time consuming. There is a misconception that if women work outside the home (much more in some settings than other, very strong here in Mexico), they are neglecting their child rearing role and their children will be negatively affective by this. Social expectations and norms are so strong, and it is impossible to be everywhere and to do everything at once.”
**Women are our own worst enemies**
“I think for me currently, the biggest challenge is not extrinsic, it is intrinsic. I do not have the self-confidence that I can be a leader in this field yet and need to change how I act to change this.” “I’ve (had) problems with other women thinking they support women in leadership but (are) actually undermining other women. It’s a topic that is rarely discussed but if women are going to make progress we have to take a long hard look at how women do and don’t support each other.”
**Additional challenges for women of color and other marginalized groups**
“Considering a woman’s intersectionality (the other traits that work in combination to create bias (ageism, racism, genderism, etc.)) is also important to address so that as we work as women we do not recreate similar inequalities as historic systems have allowed.”
**Differences in male and female leadership styles**
“I think men and male-created institutions still define what a good leader (is) with a gendered lens. As a woman leading an organization…. I am judged according to a male-centric definition of leadership.”


*Systemic/cultural factors* include broad social norms, institutional biases, and structures in place that make it difficult for women to advance. Many respondents suggested that we need to move beyond thinking about individual-level changes and focus on addressing social, cultural, and systemic issues.

“Working abroad in countries with unequal views of roles for men versus women can be very challenging. If over and over you don’t feel respected, it becomes very difficult to keep standing up for yourself.”

Similar to the quantitative data, many respondents indicated that *mentorship* was critical to overcoming gender disparities in leadership.

“I would say the biggest challenge is the innate human tendency to mentor people who remind you of yourself. I have incredible bosses and colleagues who are men, and I do think they are genuinely interested in advancing women, but there are many moments where the opportunity to name a point person, or give a young scientist a speaking slot or new responsibility and they default to thinking of a young man for the opportunity.”

The *critical role of support* for women interested in leadership roles was mentioned, including financial and non-financial support from men, from other women, and from institutions.

“I am as successful as I am today because I had men in power supporting me in front of others and noting my strengths and accomplishments for me. Without them, I wouldn’t have been given the time of day.”

*Role conflict and issues surrounding work/life balance* were also repeatedly described as a barrier to advancement for women. While these issues intersect with some of the systemic/cultural issues described previously, the issue came up frequently enough to warrant its own code. Participants from both HICs and LMICs voiced concerns.

“It seems like all the women I look up to as global health leaders are somewhat miserable because it is so challenging to both travel extensively for work and raise a family…As a young global health professional I feel a lot of pressure to choose – either to have a family and remain in a low-level role forever or decide not to have a family and pursue leadership positions.”

At the same time, some respondents suggested that the issues for women should not be anchored in the roles of wife and mother, as many women eschew such roles and still face challenges in reaching positions of leadership.

“(I’d like to see) less about being mothers, wives, etc. etc. which is alienating for hard-working delegates unable to have children, LGBTQI delegates, unmarried, those who’ve sacrificed relationships for education and careers.”

Participants disagreed over the extent to which “*we are our own worst enemies*”, with some participants suggesting that women needed to be more assertive, while others bristled at the suggestion that they should be blamed for gender inequity in leadership.

“Women in my country lack guidance and mentorship, because they don’t seek it fearing they are not good enough and having a constant fear of messing up.”

“It is not ‘my’ lack of assertiveness. Please stop blaming ‘me’. I shouldn’t have to be assertive to be treated fairly and have my expertise recognized, whether I am 5 years old, 50 years old, or 105 years old, whether I am male, transitioning, or female, regardless of my race or country of origin. It is about justice and fairness. I understand needing the skills to work in the system as it is, but do not blame me for not being assertive (because trust me, I am assertive).”

Some respondents indicated that women need to do a better job of supporting one another.

“Most women don’t like to mentor or encourage each other. Most of them have “Pull Her Down” Syndrome… never will they applaud someone’s efforts but will always give negative comments which brings someone down.”

One of the factors elucidated by the qualitative data that was not in the quantitative data related to the *additional challenges faced by women of color and others in marginalized groups*.

“I think the greatest issue is actually around women of color. We risk making the mistake of the overall western feminist movement by overlooking compound issues faced by people of different races or ethnicities or, for that matter, abilities, education, or sexual orientation.”

This extended to disciplinary biases, as well, as several nurses noted the struggle that the nursing profession has had in placing leaders in coveted global health leadership positions.

The final factor identified in the qualitative data that did not appear in the quantitative data focused on *intrinsic differences in leadership styles between men and women*.

“(There are) expectations that to be a leader you have to lead like a man (this comes from men AND women). (There is also a) lack of recognition that women’s softer skills are valuable. Instead they come across as being ‘too nice’ or ‘not enough of a leader.’”

“One of the biggest challenges is that we do not acknowledge and appreciate the different forms of leadership that are presented by women. I believe we need a shift in what we think leadership can look like.”

### Does gender bias impede career growth?

More than half of respondents, regardless of their place of residence, agreed that “I personally feel like gender bias has affected my career growth in global health” (p = 0.44, see Table 2). Multivariate logistic regression showed that the only factor significantly associated with perceiving that gender bias had impacted one’s career was being white. (See Table 4).

**Table 4:** Logistic regression analysis showing variables associated with a personal belief that gender bias has affected global health career growth.

Variable	Odds Ratio	Standard Error	P Value		95% CI

Age (<=40, ref)					
41 and above	1.3	0.30	0.24		0.82–2.07
Race (African, ref)					
Asian	2.6	1.01	0.012		1.23–5.61
Hispanic	1.2	0.76	0.700		0.38–4.12
White	2.6	0.94	0.007	*	1.29–5.31
Mixed	2.0	1.07	0.178		0.72–5.70
Other	2.8	1.91	0.113		0.77–10.58
Employment type (Academia, ref)					
Private/for-profit	0.5	0.21	0.115		0.23–1.17
Private/not-for-profit	0.9	0.26	0.975		0.58–1.67
Public sector/government	1.6	0.75	0.269		0.67–4.1
Other	0.5	0.27	0.224		0.18–1.48
Country of residence (HIC, ref)					
LMIC residence	1.8	0.57	0.069		0.95–3.36
Constant	0.6	0.19	0.097		0.28–1.11

Qualitative data suggest gender bias is a significant factor for women seeking leadership roles, but there is lack of agreement on whether it is any better or worse in global health than in other fields.

“I think global health magnifies other existing gender bias and makes it even more complex when we are working in settings of cultural, economic, social, racial differences. I never noticed a ‘glass ceiling’ in my other work but in global health there is the bias I always heard about…”

“I think global health is probably better than some fields in terms of number of women in leadership and opportunities to advance, but still gender bias/being treated differently can be hard and can lead to lower confidence.”

“It really depends on the country context as well as institution of focus, but across the board, it does appear that institutionalized gender bias is a barrier for most women working in this field, regardless of country of origin.”

It is also noteworthy that male respondents recognized the impact of gender bias on their own career trajectories.

“When I said my own career was affected by gender bias, I meant that I have probably benefitted from bias, in that some of my success is likely attributable simply to looking the part, especially early on when I had no proven track record. I probably got the benefit of the doubt that women or members of minority groups might not have gotten.”

### Proposing solutions

Our final research question focused on perceptions regarding solutions and a pathway forward. Solutions fell into two main categories: individual solutions and meta-level solutions. As one respondent said,

“We have largely focused on individual-level interventions to overcome (women’s barriers to advancement) rather than looking at how we can change organizations, systems, and policies to better overcome these barriers. We need systemic interventions if we are going to make an impact.”

At the same time, women describe needing tangible skills to help them navigate the challenges they face every day. “How should I talk to these demeaning superiors that at once puts them in their place but also doesn’t leave them feeling alienated and threatened (and gets me fired?)”

For every barrier to advancement described above, respondents provided a variety of suggested solutions that ranged from individual-level to institutional or societal-level actions. For example, with regard to mentorship, respondents suggested that while senior women ought to seek out junior counterparts to mentor and include in high-level activities to facilitate their transition to positions of leadership, junior women ought to pro-actively seek out senior-level mentors and make their desire for leadership known. On an institutional level, incentivizing mentorship and increasing recruitment of women ought to be prioritized. Table 5 summarizes the most common solutions provided.

**Table 5:** Barriers to Leadership with Suggested Solutions.

Barrier	Individual-level solution	Meta-level solution

Common to both HIC and LMIC respondents		

Lack of mentorship/sponsorship	- ** Reach out:** Senior level women reach out to junior women; include them on committees and invite them to important meetings - ** Be pro-active:** Junior women seek out mentors and make desire for mentorship and leadership known	- ** Incentivize mentorship** at the institutional level - ** Increase recruitment of women** into training programs where they can be connected with mentors in their fields, and in turn mentor other women
Balancing work and home	- ** Ask for help:** Women refuse to accept sole responsibility of raising children and keeping the house: ask for help - ** Men (and families) step up** and provide women with more support at home and at work	- ** Targeted funding for travel:** More funding available to support women leaders traveling with their children - ** Family-friendly policies:** Policies that promote family-friendly work environments - ** Structural fixes** (e.g. childcare at conferences, delayed tenure clock, funding for maternity leave)
System/culture/gender bias	- ** Lead with courage:** Women leaders refusing to adopt a male leadership style, but instead re-defining what it means to be a leader: “I am, as a leader, less interested in moving up (a) ladder than creating a larger and more collaborative table.” - ** Increase visibility:** Nominating female colleagues for prestigious awards to help establish legitimacy - ** Speaking out** about potential for bias, advocating for unconscious bias training in the workplace	- ** Blinded recruitment** and selection of finalists without revealing gender - ** Increasing diversity on multiple axes**, not just gender - ** Instituting requirements** for percent of leaders that are female - ** Support for unconscious bias training** in the workplace
Women’s lack of assertiveness	- ** Believe!** Women need to increase their confidence and believe in themselves - “**Coaching** to help women recognize and overcome that little voice that says ‘I’m not good enough.”	- ** Leadership training** courses designed for women - ** Targeted Recruitment:** Explicit solicitation of female applicants for leadership positions: “Women are less likely to apply for jobs unless they meet 100% of the requirements, versus men who will apply even if they meet 60% of the requirements… (Some organizations) now actively reach out to qualified women who might not otherwise apply and ask them to interview without waiting for them to apply. They do this because they also know that having more women in the applicant pool is beneficial.”
**More pronounced among LMIC respondents**		

Lack of opportunities	- ** Be persistent and creative:** Make known what you want, and ask everyone in your network if they can help you make it happen. Many opportunities are created rather than advertised. - ** Look out:** Watch for leadership, communication and negotiation skills workshops; apply for scholarships to attend	- ** Open the door:** Institutions need to seek out and consider non-traditional candidates, looking beyond finding a candidate that feels familiar - ** Be creative:** Organizations can think about ways to send two employees instead of one to a meeting or workshop, or invite outside experts in for networking events rather than expecting employees to travel
Lack of funding for meetings/networking	- ** Capitalize:** Never miss an opportunity to introduce yourself/meet with leaders in the field; network locally; keep up with contacts electronically and via phone meetings - ** Prioritize:** Ask for or put aside your own professional development budget to participate in one or two key annual conferences or meetings	- ** Leverage:** Institutions can offer small grants or matching funds for conference travel, can petition conferences for LMIC scholarships - ** Sponsor local, inclusive networking events:** Organizations can sponsor local networking events that do not focus on ‘male’ zones such as bars or sporting events and do not occur late at night
Safety concerns	- ** Partner:** Travel with a group, find partners to accompany you, identify on-the-ground safety allies - ** Be Aware** of the potential safety issues, work to avoid most dangerous areas/times of day	- ** Protect your investment:** Organizations can budget for security personnel to accompany all employees – not just women – into the field - ** Be clear** about sexual harassment and discrimination policies and the pathway for grievances as well as the mechanisms of accountability
Travel requirements	- ** Pace yourself:** Identify which travel is essential and which is optional - ** Use your networks:** Identify resources to help with non-work responsibilities while you travel	- ** Invest in technology:** Organizations can explore the use of telecommuting and virtual meeting technologies, even in low-bandwidth settings, using such platforms as WhatsApp and GoToMeeting - ** Discuss and revisit what constitutes essential and non-essential travel**, being aware of the risk of over-valuing the employee who says yes to every trip.

## Discussion

The survey represents nearly 400 women and a score of men who are thinking about the issues of women’s advancement in global health, wrestling with their complexity, and seeking a better way forward. This study found that, regardless of where women live and work and regardless of their age or stage of career, most agree that women face unique barriers to advancing to positions of leadership in global health. The types of barriers cited, including lack of mentorship, balancing work and home, and gender bias, are also common across respondents. However, LMIC respondents report lack of opportunities, financial constraints, and safety concerns more often than their HIC counterparts. In multivariate analysis, being Caucasian was the only factor that predicted a positive response to a perception that gender bias has affected career growth. This may have to do with white women’s inherent privilege, allowing them to focus on gender as the main axis of bias, whereas women of color and other marginalized groups may have many other axes of bias that they perceive to have held them back. This supposition was borne out in the qualitative data, which found that women of color and other marginalized groups face a double burden – a finding we would have missed had we focused solely on quantitative data. Qualitative data also suggested that women and men have different leadership styles and the male model is seen as normative. One respondent said that as a leader, she was less interested in climbing a ladder than making the table bigger – which speaks to the crux of the difference in leadership styles. As a society, we are likely to benefit if we broaden our definition and mental model of what a good leader looks like.

Despite the many barriers described, respondents offered a variety of creative solutions. Solutions range from individual-level suggestions (e.g. nominate female colleagues for awards) to more meta-level solutions (e.g. implement blinded recruitment policies) – yet there is overall agreement that solutions need to be implemented at all levels if we are to see significant changes. Future research is warranted that not only identifies key metrics of success and measures improvement over time, but assesses the impact of various interventions in contributing to that improvement. The 2017 Women Leaders in Global Health Conference served as a catalyst for important conversations about these issues [10], but future research is needed to determine how we can translate conversations into tangible improvements.

There are several limitations to this research. First, our sample is predominantly white (50.1%) and overrepresented by those from HICs. While LMIC voices were heard through the qualitative data, their numbers and they type of respondents they likely were (English-speaking respondents who found their way to a conference based in the United States) may have affected the quantitative findings. Similarly, there were very few men in the sample, and thus we have few solutions offered by men. This research also reflects a convenience sample of those who expressed an interest in women’s leadership in global health, and were motivated enough to complete an online survey. Thus the findings cannot be widely generalized. However, by virtue of their interest and familiarity with this topic, these individuals may provide more thoughtful reflections about both the origin of gender disparity and the ultimate solution. The final limitation is related to the fact that our study design included open-ended questions to generate qualitative data, not allowing for additional probes or supplemental interviews. However, the volume and breadth of responses provided yielded a wealth of information that suggests thematic saturation had been reached.

This study is the first of its kind to attempt to quantify both the barriers to advancement for women leaders in global health, but also the potential solutions. While achieving gender parity will not be easy, we believe there is room for optimism. Individual women and men are motivated to make room at the table to ensure diversity of leadership styles and representation. Institutions can incentivize mentorship, create inclusive workplaces that recognize female leadership styles, and commit to targeted recruitment to find more diverse leaders and more female leaders. These interventions should take into account the unique challenges and opportunities of field work and the pressures they may impose on women, particularly those from LMICs. We believe that a new leadership paradigm that values diversity of thought and diversity of experience will benefit not only women aspiring to global health leadership positions, but ultimately it will further the causes to which both men and women in global health are committed.

## Additional File

The additional file for this article can be found as follows:

10.29024/aogh.2384.s1 Appendix 1. Survey Questions.
