# Supplementary material for: Advancing Women Leaders in Global Health: Getting to Solutions
Source: Ann Glob Health. 2018 Nov 5;84(4):743–52. doi: 10.29024/aogh.2384 (PMC6748298; doi:10.29024/aogh.2384)
Supplement: Appendix 1. — Survey Questions. [file agh-84-4-2384-s1.pdf]

Appendix 1: Survey Questions

1. My age is…
2. I identify my gender as… Female, Male, Trans
3. My country of origin is…
4. My current country of residence is…
5. I would describe myself as… African, Asian, Hispanic, Native American, White, Mixed, Other
6. My current marital status is best described as…. Married / domestic partnership, single (never married), divorced/widowed, separated
7. I have ___ dependents currently living with me or for whom I am a primary care giver.
8. I would describe myself as…. Working full-time, working part-time, not currently working
9. I consider myself… early career, mid-career, late career
10. My current (or most recent) position is best described as… academia, private / for-profit, private / not-for-profit, public sector / government, other
11. Which best describes you? I am in a position of global health leadership, I aspire to a position of global health leadership, I do not aspire to a leadership role
12. Did you attend any portion of the Women Leaders in Global Health Conference at Stanford Oct 11–12, either in person or via livestream? Yes, attended in person; Yes, attended via livestream; No (skip to Q20)
13. How would you rate the Women Leaders in Global Health conference overall? Poor, fair, good, very good, excellent
14. How would you rate the Women Leaders in Global Health conference in terms of learning opportunities? Poor, fair, good, very good, excellent
15. How would you rate the Women Leaders in Global Health conference in terms of networking and collaboration opportunities? Poor, fair, good, very good, excellent
16. How would you rate the Women Leaders in Global Health conference in terms of leadership-building opportunities? Poor, fair, good, very good, excellent
17. How likely are you to recommend a future Women Leaders in Global Health Conference to a friend or colleague? Extremely unlikely, somewhat unlikely, neither unlikely nor likely, somewhat likely, extremely likely
18. What would you say are some of the main messages (or action steps) you took away from the conference?
19. What might you recommend for next year's Women Leaders in Global Health Conference to be held in London? (e.g. Speakers? Topics? Format? Duration? Role of men?)

Now we have a few questions about your thoughts, attitudes and beliefs regarding some of the issues in global health leadership for women. How do you feel about the following statements:

20. Women face unique barriers to advancing to positions of global health leadership when compared to men. Strongly disagree, somewhat disagree, neither agree nor disagree, somewhat agree, strongly agree

21. I personally feel like gender bias has affected my career growth in global health. Strongly disagree, somewhat disagree, neither agree nor disagree, somewhat agree, strongly agree
22. Looking at the list below, please indicate the factors that you think are the most significant barriers for women in global health. (Not important, Somewhat important, Most important (pick 3))
    a. Lack of mentorship / sponsorship
    b. Lack of female mentors / role models
    c. Lack of opportunities / experience
    d. Lack of training
    e. Lack of assertiveness / confidence
    f. Gender bias in home country / institution (implicit or explicit)
    g. Gender bias in partner country / institution (implicit or explicit)
    h. Travel requirements
    i. Work load
    j. Balancing work and family
    k. Inadequate pay
    l. Safety concerns
23. These next few questions move away from the short answer format of above. We are hoping you can take a few minutes to respond and help us understand some of the bigger issues in women's leadership in global health. (If you prefer to think about these questions and answer later, you can email qualitative responses to (email address), but this will waive your anonymity.) First, what would you say is the biggest challenge facing women either in positions of leadership in global health or aspiring to positions of leadership in global health today?
24. What is one of the biggest challenges FOR YOU in this area, if you have not already addressed it in the previous question?
25. Can you describe any interventions, programs, or situations that you think successfully enable(d) women to rise to positions of leadership in global health?
26. Thank you for your time, and please feel free to leave any additional comments you may have in the space below.
